# The human blood parasite *Schistosoma mansoni* expresses extracellular tegumental calpains that cleave the blood clotting protein fibronectin

**DOI:** 10.1038/s41598-017-13141-5

**Published:** 2017-10-10

**Authors:** Qiang Wang, Akram A. Da’dara, Patrick J. Skelly

**Affiliations:** Molecular Helminthology Laboratory, Department of Infectious Disease and Global Health, Cummings School of Veterinary Medicine, Tufts University, North Grafton, MA 01536 USA

## Abstract

Schistosomes are intravascular, parasitic flatworms that cause debilitating disease afflicting >200 million people. Proteins expressed at the host-parasite interface likely play key roles in modifying the worm’s local environment to ensure parasite survival. Proteomic analysis reveals that two proteases belonging to the calpain family (SmCalp1 and SmCalp2) are expressed in the *Schistosoma mansoni* tegument. We have cloned both; while highly conserved in domain organization they display just 31% amino acid sequence identity. Both display high relative expression in the parasite’s intravascular life forms. Immunolocalization and activity based protein profiling experiments confirm the presence of the enzymes at the host-parasite interface. Living parasites exhibit surface calpain activity that is blocked in the absence of calcium and in the presence of calpain inhibitors (E64c, PD 150606 and calpastatin). While calpains are invariably reported to be exclusively intracellular (except in diseased or injured tissues), our data show that schistosomes display unique, constitutive, functional extracellular calpain activity. Furthermore we show that the worms are capable of cleaving the host blood clotting protein fibronectin and that this activity can be inhibited by E64c. We hypothesize that SmCalp1 and/or SmCalp2 perform this cleavage function to impede blood clot formation around the worms *in vivo*.

## Introduction

Schistosomes are parasitic flatworms that live in the vascular system of vertebrates, causing a chronic, debilitating disease that afflicts over 200 million people in over 70 countries^1^. Another 800 million people are at risk of infection^2^. The disease caused - schistosomiasis - ranks as the second most socioeconomically devastating parasitic disease in the world. Annual mortality is estimated at over 250,000 in sub-Saharan Africa alone, with many more experiencing chronic morbidity^3,4^. There are three major species that infect humans: *Schistosoma mansoni, S. japonicum and S. haematobium*, of which *Schistosoma mansoni* has the widest geographical distribution. Infection occurs when the free-swimming larval form (the cercaria) penetrates the skin and transforms into a morphologically and biochemically distinct life stage called the schistosomulum. This juvenile parasite invades a blood vessel, migrates and matures. Adult schistosomes live as pairs in the mesenteric veins or the vesicle venous plexus, sometimes for years^1^.

Schistosomes in the bloodstream are surrounded by components of the immune system and the hemostatic system, yet remain able to survive without causing overt inflammation or thrombus formation around them^5–9^. This means that they must possess evasion strategies that can overcome these two host defensive systems. We hypothesize that the parasite tegument (skin), specifically the host-interactive surface, provides protection for the worms, and we are investigating the molecular capabilities of selected proteins expressed at the worm surface. We hypothesize that these proteins play key roles in modifying the local environment of the worms to ensure their survival. Some surface proteins are important for nutrient uptake^10,11^ or water exchange^12^ and some may exert an anti-inflammatory^13^ or anti-thrombotic effect^7,14^.

Among the proteins detected in proteomic studies of schistosome surface membranes are two that belong to the calpain family. Calpains (EC 3.4.22.17; Clan CA, family C02) constitute a distinct group of cysteine proteases found in almost all eukaryotes and a few bacteria. They are defined as cytosolic enzymes exhibiting Ca^2+^-dependent proteolytic activity at a neutral pH^15,16^. Calpains are not considered degradative enzymes, but instead they engage in limited cleavage of target proteins in response to calcium signalling^17^.

A large number of proteins (over 100) can be cleaved by different calpains in *in vitro* assays including cytoskeletal proteins, enzymes, receptors, ion-channel proteins and transcription factors^18^. From this diversity of substrates, it is clear that calpains can be involved in a diversity of molecular functions including cell motility, signal transduction, assembly of focal adhesions, cell cycle regulation and apoptosis. Humans possess 14 calpains and disturbances in expression of these proteins have been associated with a number of pathological conditions, including muscular dystrophy, ischemia, stroke and brain trauma, various platelet syndromes, hypertension, liver dysfunction, and cancer^16,19,20^.

“Classical” calpains have a conserved domain organization first described in the human calpains CAPN1 and CAPN2; they contain a proteolytic domain followed by a calpain-type beta-sandwich (CBSW) domain important for phospholipid binding (and previously known as the C2-domain-like (C2L) domain) and a carboxyl-terminal, Ca^2+^-binding, helix-loop-helix, penta-EF-hand (PEF) domain. The *Schistosoma mansoni* genome encodes five homologs belonging to this classical calpain family^15^ and two of these (SmCalp1 and SmCalp2, the focus of this work) have been detected in the host-interactive tegumental membranes of the intravascular parasites by proteomic analysis^21–23^. The notion that schistosomes express functional calpains that could interact with extracellular host proteins is intriguing since calpains are invariably reported to be located exclusively intracellularly (except in diseased or injured tissues)^18^.

While no work has previously been conducted on SmCalp2, SmCalp1 was earlier identified as being a target of natural humoral immunity; a pool of sera from *S. mansoni*-infected individuals contained antibodies that recognized this protein and led to its cDNA being cloned^24^. Simultaneously, a second research group also cloned the SmCalp1 cDNA after probing an expression library with polyclonal antiserum raised against purified *S. mansoni* membranes^25^. SmCalp1 has been tested as a vaccine candidate (called Sm-p80) in a variety of formulations and has been found to be mostly protective^26–28^. Similarly, the *Schistosoma japonicum* homolog – SjCalp1 – has been cloned and tested in vaccine trials where it has been found to be generally protective^29,30^. While these vaccine trials suggest the SmCalp1 is host exposed, they do not reveal if the parasites express a functional extracellular calpain. In this work we set out to clone both of the *S. mansoni* tegumental calpains, SmCalp1 and SmCalp2 and to determine if living worms do possess an external calpain activity. We find that all intravascular life stages can cleave a non-cell-permeable calpain substrate in a Ca^2+^-dependent manner and this activity can be inhibited by known calpain inhibitors. Further, we show here that one substrate of this activity is the key blood-clotting protein fibronectin. We propose that schistosome calpain-mediated cleavage of fibronectin could limit firm blood clot formation around the worms *in vivo* to permit the parasites more unrestricted movement in their intravascular niche.

## Results

### Schistosoma mansoni tegumental calpains

Proteomic studies revealed the presence of two schistosome calpain homologs in the tegument of *Schistosoma mansoni* ^21–23^. We designate these two calpains as SmCalp1 and SmCalp2. In the schistosome DNA sequence database, GeneDB, SmCalp1 (also known as Sm-p80) is annotated as Smp_214190 (previously part of Smp_157500). The coding sequence of this protein has been published^24,25^; it has a predicted molecular weight of 86,920 Da and its predicted isoelectric point (pI) is 5.24. SmCalp2, annotated in GeneDB as Smp_137410, has not been investigated before. As described in Methods, we used proteomic and genomic data, to design primers which were used to amplify the cDNAs encoding SmCalp1 and SmCalp2. These were sequenced at the Tufts University Core Facility. The predicted molecular weight of the SmCalp2 protein is 90,902 Da and its predicted pI is 7.6. The SmCalp2 cDNA sequence reported here contains an additional 54 amino acids at the N-terminus as well as an additional internal 10 amino acids (I^340^ - G^349^) compared to the currently annotated sequence at NCBI (accession number XP_018648578). Supplementary Figure S1 shows an alignment of SmCalp1 and SmCalp2.

**Figure 1:**
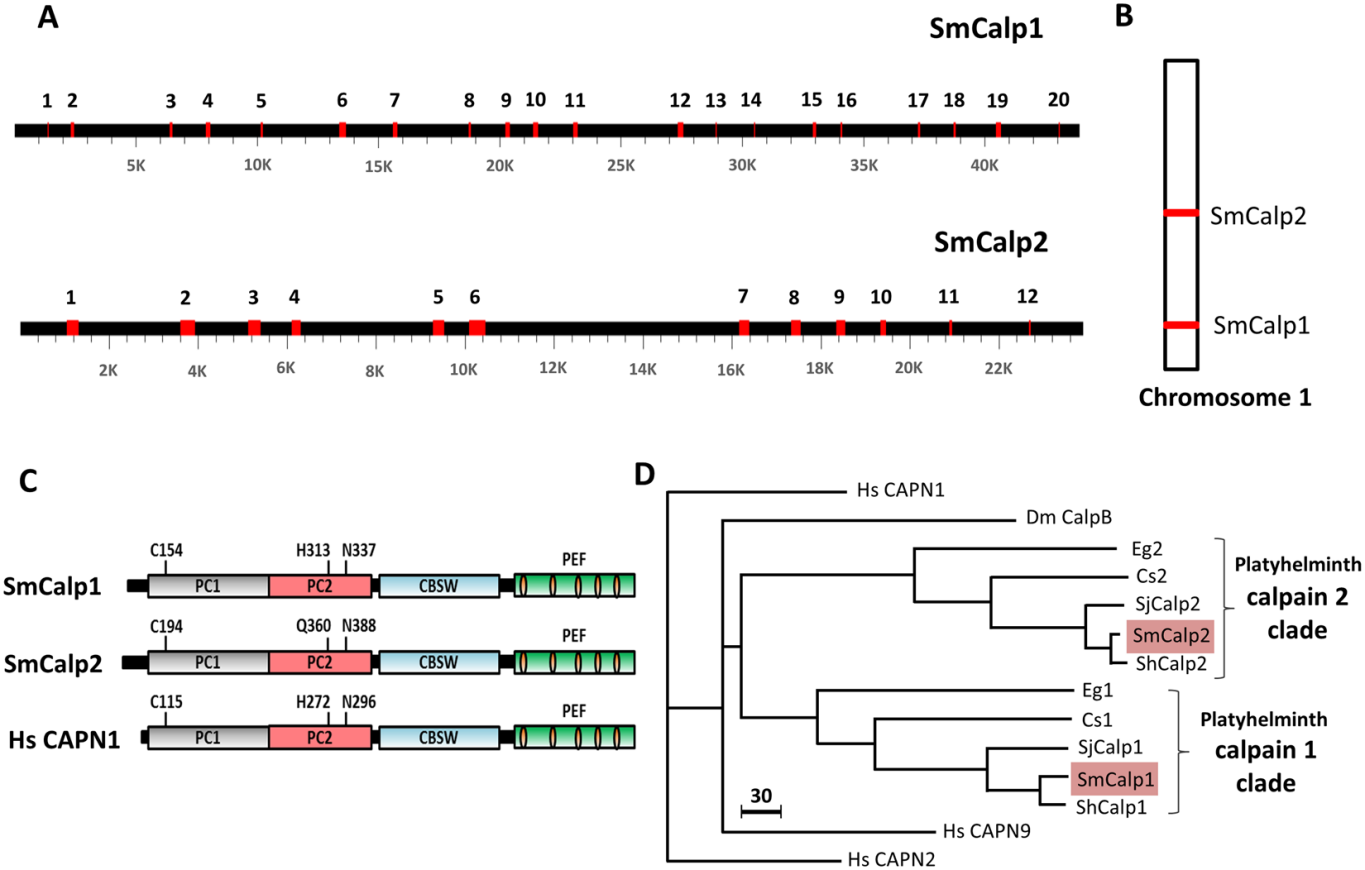
SmCalp1 and SmCalp2 gene and protein organization. (**A**) Diagrammatic representation of the gene for SmCalp1 (top) and SmCalp2 (bottom). Exons are depicted in red and are numbered above each gene. Scale numbers below each gene represent kilobases (K). (**B**) Diagrammatic representation of *S. mansoni* chromosome 1 showing the location of the genes for SmCalp1 and SmCalp2. (**C**) Domain structure of SmCalp1, SmCalp2 and human calpain CAPN1. PC1 (grey) and PC2 (red) represent the protease core domain; CBSW domain is depicted in light blue and the penta-EF hand domain in green (containing five helix-loop-helix motifs (orange)). The letters and numbers on top of each PC1 and PC2 domain represent the positions of conserved active site amino acid residues. (**D**) Unrooted phylogenetic tree of selected calpains generated by neighbor joining with Accelrys Gene software. The scale bar represents the number of amino acid differences per unit length. Designations (and accession numbers) are as follows: *S. mansoni* Calp1 (SmCalp1; AAA29857.1); *S. haematobium* Calp 1 (ShCalp1, BAF62290.1); *S. japonicum* Calp1 (SjCalp1, BAA74718.1); *Clonorchis sinensis* Calp 1 (Cs1, GAA52477.1); *Echinococcus granulosus* Calp1 (Eg1, CDS23506.1); *S. mansoni* Calp2 (SmCalp2; MF590064)**;** *S. haematobium* Calp2 (ShCalp2, XP_012791984.1); *S. japonicum* Calp2 (SjCalp2, MF590065)**;** *Clonorchis sinensis* Calp2 (Cs2, GAA295577.2); *Echinococcus granulosus* Calp2 (Eg2, CDS20315.1); *Drosophila melanogaster* CalpB (Dm CalpB, NP_524016.4); Human calpain 9 (Hs CAPN9, NP_006606); Human calpain 2 (Hs CAPN2, NP_001739), Human calpain 1 (Hs CAPN1, NP_005177).

Using the available *S. mansoni* genome sequence at GeneDB.org and at Parasite.WormBase.org, the genes for both SmCalp1 and SmCalp2 were identified. Figure 1A depicts the exon/intron organization of the two genes; SmCalp1 has 20 exons (shown in red) and extends over 40 kb while SmCalp2 contains 12 exons and is ~22 kb in size. Both genes are located in chromosome 1 (whose current estimated size is 65,476,681 bp) as shown in Fig. 1B; the SmCalp2 gene is found towards the middle of chromosome 1 (position: 33,185,000–33,207,000) while SmCalp1 is more distally located (position: 57,382,000–57,424,000). The domain structure of SmCalp1 and SmCalp2 is depicted in Fig. 1C and is compared with the domain structure of the human classical calpain, CAPN1. Figure 1C shows that both SmCalp1 and SmCalp2, like CAPN1, are predicted to contain all of the domains found in members of this classical calpain family^24^. PC1 and PC2 are the protease core domains 1 and 2 (grey and red in Fig. 1C) which contain conserved active site residues. SmCalp1 has the same conserved residues in its catalytic domain as most classical calpains; they are C^154^, H^313^ and N^337^. Whereas in SmCalp2 the conserved active site histidine (H^313^ in SmCalp1) is replaced with glutamine (Q^360^ in SmCalp2). All members of the platyhelminth Calp2 clade (described below, including SjCalp2 and ShCalp2) share a glutamine (Q) at this site. CBSW (light blue in Fig. 1C) represents the calpain-type beta-sandwich region containing a basic loop and an acidic loop, involved in protein/cell membrane interaction^31^. The penta EF (PEF) hand domain (green in Fig. 1C) contains five Ca^2+^-binding helix-loop-helix structural domains (orange) at the C-terminus. Conservation of all of these domains in SmCalp1 and SmCalp2 clearly place both proteins in the classical calpain family.

While both schistosome calpains exhibit great structural domain conservation as just described, at the amino acid level they are quite divergent, displaying just 31% amino acid sequence identity. Sequence comparisons are shown in supplementary Figure S1. As shown in Fig. 1D, phylogenetic analysis demonstrates that both SmCalp1 and SmCalp2 (red boxes) belong to their own specific platyhelminth calpain clades. For example, SmCalp1 has highest sequence identity with homologs from the other human schistosome species, *S. haematobium* (95% identity) and *S. japonicum* (84% identity). Homologs from the trematode *Clonorchis sinensis* and the cestode *Echinococcus granulosus* also tree closely with the schistosome SmCalp1 group (with 63% and 50% identify, respectively). In a similar vein, SmCalp2 displays closest similarity with schistosome homologs ShCalp2 (98% identity) and SjCalp2 (92% identity), with homologs from the platyhelminths *C. sinensis* and *E. granulosus* more distant. Sequence comparisons of the platyhelminth Calp2 clade proteins are shown in supplementary Figure S2. Both SmCalp1 and SmCalp2 are clearly distant both from each other and from the *Drosophila melanogaster* calpain CalpB as well as the human calpains CAPN1, CAPN2 and CAPN9.

### The *S. mansoni* tegument external surface has functional calpain

#### Immunolocalization of schistosome surface calpains

**Figure 2:**
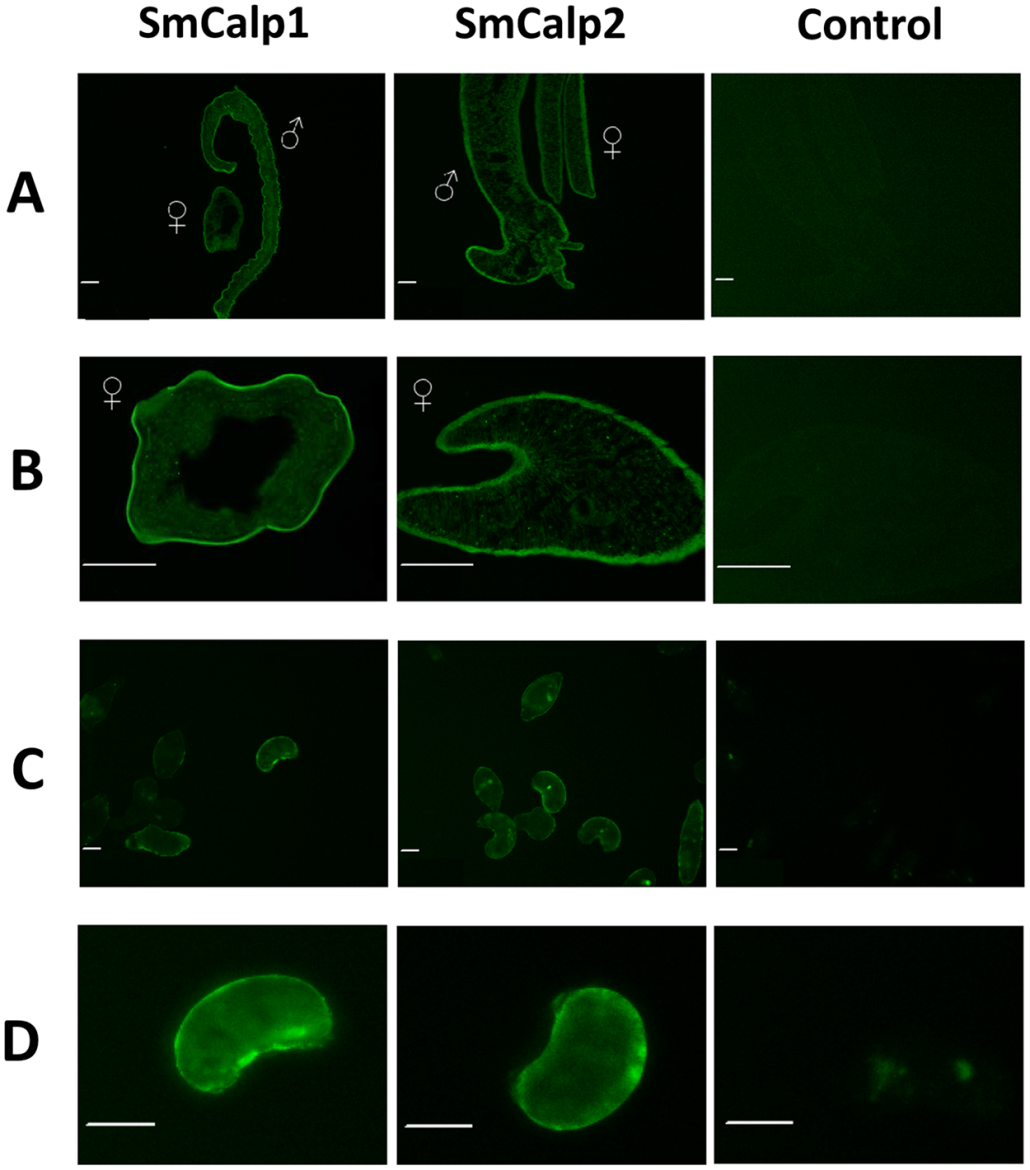
Immunolocalization of SmCalp1 and SmCalp2 in different *S. mansoni* life stages. (**A**) Longitudinal section of adult male and female parasites. (**B**) Cross section of adult female parasites. Scale bar in **A** and **B** represents 50 µm. (**C**,**D**) Whole 7-day cultured schistosomula; scale bar represents 25 µm. Preparations were probed with anti-SmCalp1 antibodies (left panel), anti-SmCalp2 antibodies (center panel) or no primary antibody (Control, right panel).

Anti-SmCalp1 and anti-SmCalp2 antibodies were used to immunolocalize SmCalp1 and SmCalp2 in *S. mansoni* adult sections and in whole, 7-day cultured schistosomula. Figure 2 shows strong SmCalp1 (left panel) and SmCalp2 (central panel) staining predominantly in the tegument in all cases. Images of adult male and female parasites in longitudinal section are shown in Fig. 2A, cross sections of females are shown in Fig. 2B, and whole 7-day cultured schistosomula are shown in Figs 2C and 2D (at higher magnification). A clear “green ring” of tegumental staining around the parasites is revealed especially in the case of the adults. At this resolution, there is no obvious difference between the localization of SmCalp1 versus SmCalp2. Control parasites, exposed to secondary antibody alone (Fig. 2, right panel), do not display signal in the tegument or elsewhere in either adult parasites or schistosomula.

### Calpain enzymatic activity assay

**Figure 3:**
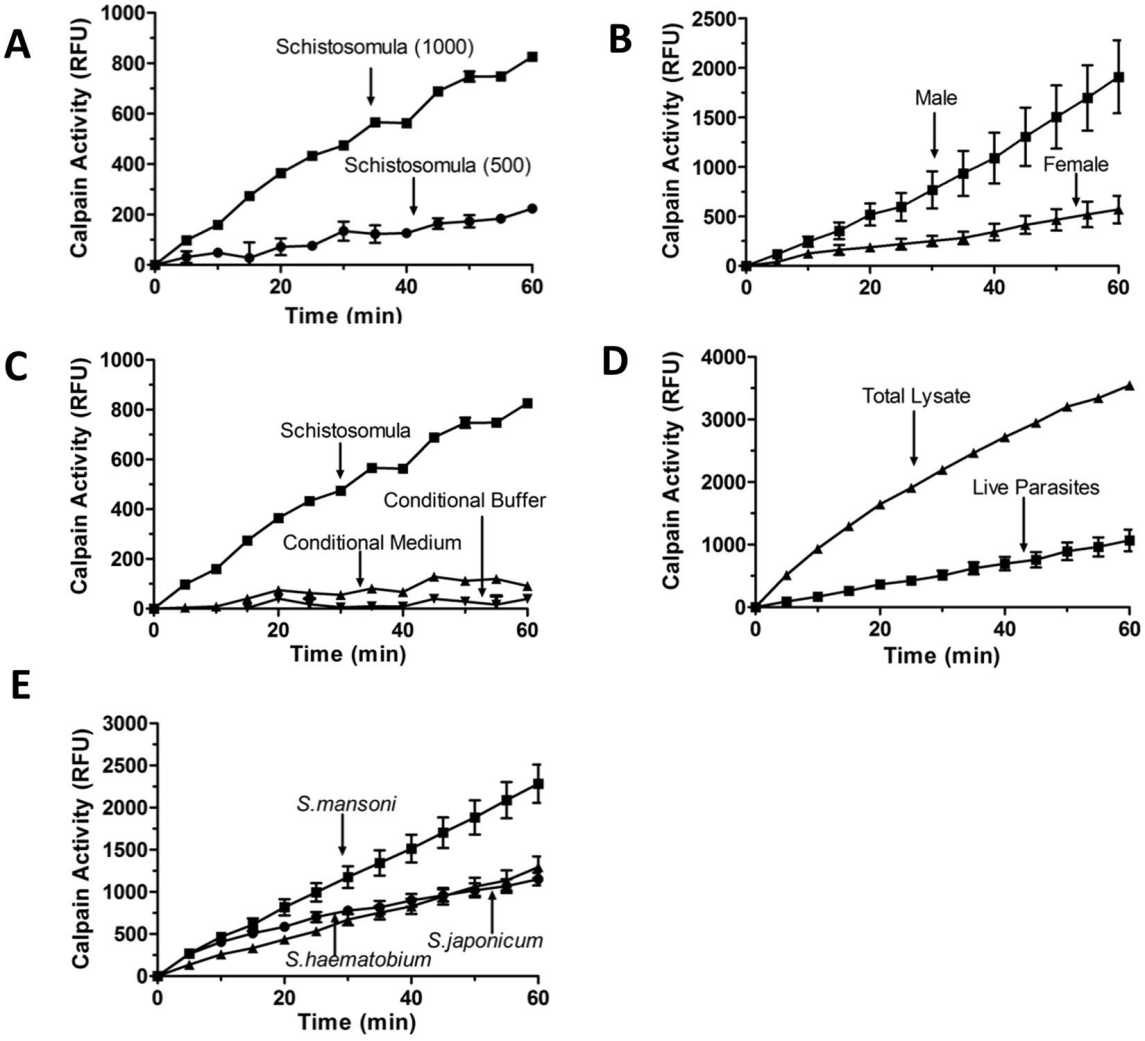
Measuring calpain activity in living schistosomes. (**A**) Calpain activity detected in live schistosomula (500 or 1000, as indicated) over time. (**B**) Calpain activity in living individual adult male (square,>10 individuals) and female (triangle,>10 individuals) parasites over time. (**C**) Calpain activity in 1000 live schistosomula (squares, groups of >5), compared to that seen in either conditional buffer (i.e. buffer that had contained 1,000 schistosomula for 1 h, up triangles) or in conditional medium (i.e. medium that had contained 1,000 schistosomula for 3 days, down triangles). (**D**) Calpain activity detected in individual living adult male parasites (squares) compared to that detected in total lysates of individual males (triangles). (**E**) Calpain activity in individual adult males of *Schistosoma mansoni*, *S. japonicum*, and *S. haematobium* (as indicated). All data are presented as relative fluorescence units (RFU, mean+/−SD, n ≥ 3). Calpain activity is given in relative fluorescence units (RFU). Fluorescence is generated following substrate cleavage and is measured at excitation/emission of 320/480.

After confirming the presence of calpains in the tegument of intravascular life stage schistosomes, we investigated whether the parasites express functional extracellular calpain enzyme activity at the host-parasite interface. In order to determine this, the ability of living parasites to cleave the membrane non-permeable, fluorogenic calpain substrate “Calpain Substrate III” (Calbiochem) was measured. Using this assay, calpain activity was detected in live schistosomula and live adult parasites. As shown in Fig. 3A, calpain activity increases over time as the number of schistosomula included in the experiment increases. Similarly, Fig. 3B shows that calpain activity increases over time for both male and female worms with individual males displaying higher calpain activity compared to individual females. Since the calpain substrate used is cell impermeable, the activity detected derives from extracellular enzyme associated with the surface of the worms.

To investigate the possibility that calpain is released or secreted by cultured parasites, we performed an experiment in which ~1000 schistosomula were first incubated in assay buffer. After 1 hour the buffer was recovered and any calpain activity in the buffer was measured. Separately, complete medium in which 1000 schistosomula had been cultured for 3 days was collected and calpain activity therein was measured. As a positive control, a standard calpain activity assay was conducted using 1000 live schistosomula. Figure 3C shows the results of these analyses which demonstrate that essentially no calpain activity is detected in either the conditioned buffer or in conditioned medium (Fig. 3C, lower lines). Only buffer containing parasites exhibits clear calpain activity (Fig. 3C, upper line (squares)) showing that enzyme remains associated with the external surface of the parasites and, at least within the time frame examined, is not released.

In Fig. 3D, the surface calpain activity is compared with the total calpain activity detected in a homogenate of a single male. This experiment shows that the surface calpain activity makes up about 30% of the total detectable. Finally, live, single male worms of the three major schistosome species that infect humans: *S. mansoni, S. haematobium* and *S. japonicum* were compared for surface calpain activity and Fig. 3E shows the results. Extracellular calpain activity is detected in each case with the highest level being seen in *S. mansoni*.

### Characterizing live schistosome tegumental calpain activity

**Figure 4:**
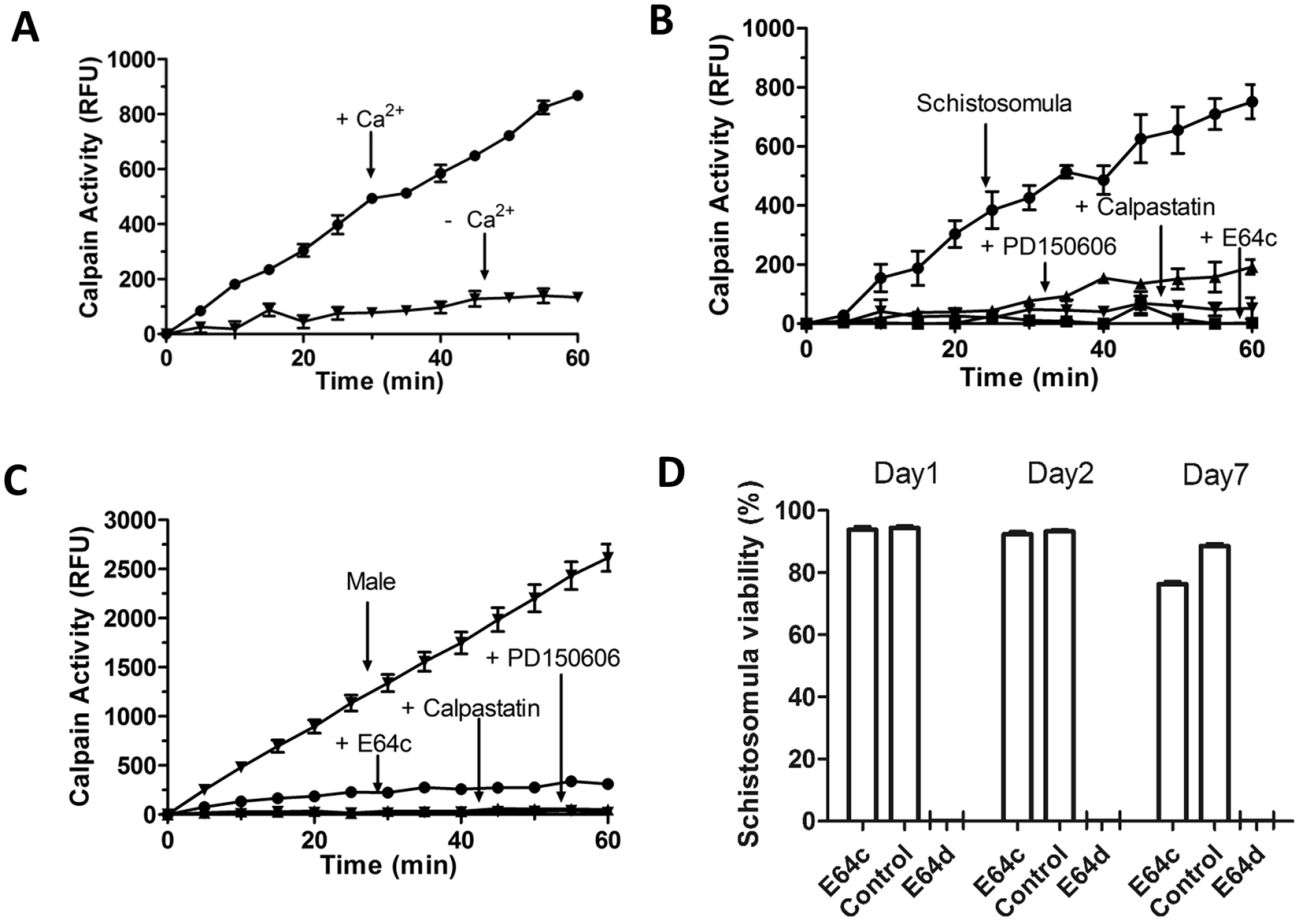
Characterization of schistosome surface calpain activity. (**A**) Calpain activity measured in 1000 live schistosomula in the presence ( + ) or absence (−) of 3 mM Ca^2+^. Calpain activity detected in 1,000 living schistosomula (**B**) or individual male worms (**C**) in the presence or absence of the calpain inhibitors (E64c, calpastatin and PD150606, as indicated). Parasites were pre-incubated with inhibitor for 20 min before the addition of substrate. All activity data are presented as relative fluorescence units (RFU, mean+/−SD, n ≥ 3) where fluorescence is generated following substrate cleavage and is measured at excitation/emission 320/480. (**D**) Analysis of schistosomula viability after incubation in the presence of inhibitor (E64c or E64d, 100 µM) for 1, 2 or 7 days (as indicated). Data are shown relative to day 0, control parasite viability, set at 100%.

To determine if the enzymatic activity exhibited by living parasites requires Ca^2+^ (as is characteristic of other calpain enzymes^32^), the activity assay was conducted with living worms in the presence or absence of Ca^2+^. Figure 4A shows that removing Ca^2+^ from the assay buffer effectively shuts down activity (p < 0.0001).

Similarly, to determine if the enzymatic activity exhibited by living parasites could be blocked by known calpain inhibitors, worms were incubated with the membrane non-permeable inhibitor E64c or the permeable inhibitors calpastatin or PD150606. The calpain activity displayed by living schistosomula (Fig. 4B) and adult males (Fig. 4C) is effectively blocked by the presence of any of these inhibitors (p < 0.001). Worms treated in culture for 1–2 days with E64c exhibit no morphological differences compared to controls. However, after prolonged incubation in the presence of E64c (7 days), treated worms show ~10% lower viability compared to untreated controls (p < 0.001). In contrast, incubation of parasites with a membrane-permeable form of E64c (known as E64d) results in 100% worm killing within 24 h (Fig. 4C).

### SmCalp1 and SmCalp2 are highly expressed in intravascular life stages

**Figure 5:**
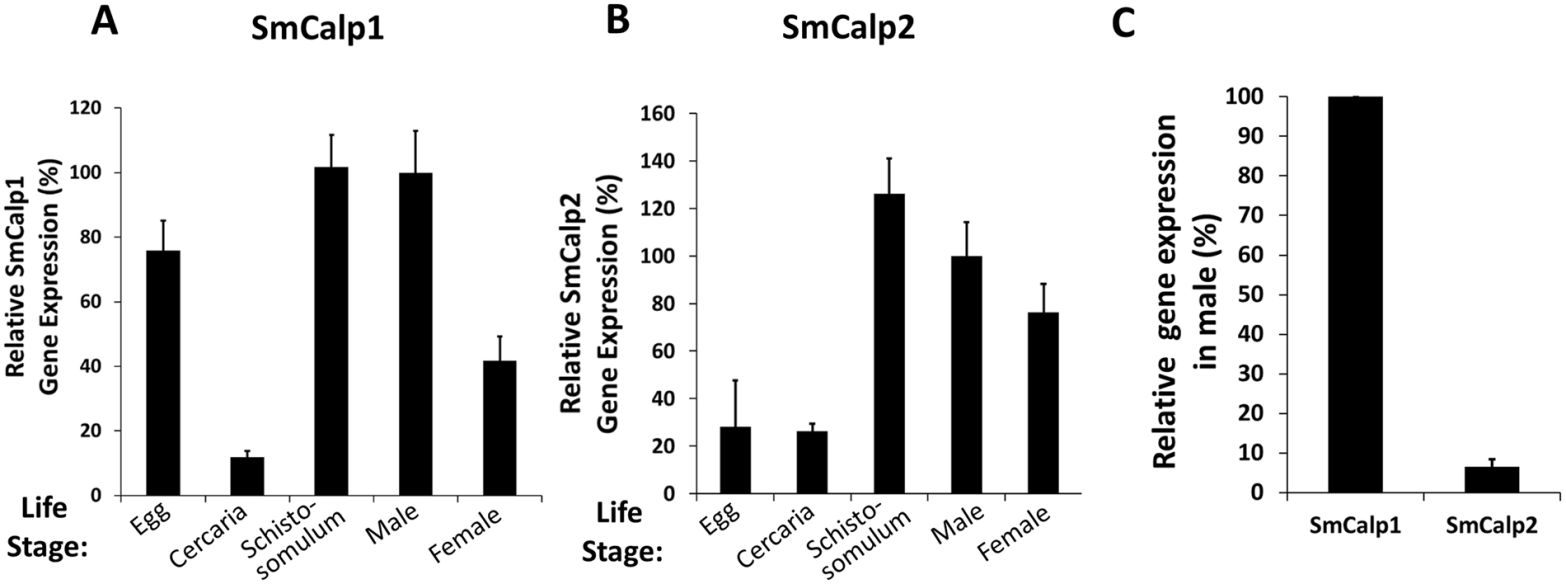
Expression of SmCalp1 and SmCalp2 in different *S. mansoni* life stages. Relative gene expression of SmCalp1 (**A**) and SmCalp2 (**B**) in the life stages indicated. All values are relative to males (set at 100%) (Mean+/− SD, n = 3). (**C**) Relative gene expression of SmCalp1 versus SmCalp2 in adult males (Mean+/− SD, n = 4). SmCalp1 gene expression is significantly higher than that of SmCalp2 (p < 0.0001, Students t test).

The relative expression of SmCalp1 and SmCalp2 in different schistosome life stages was measured using RT-qPCR and Fig. 5 shows the results of this analysis. Both SmCalp1 (A) and SmCalp2 (B) are relatively highly expressed in intravascular life stages particularly in schistosomula and males. For both genes, lowest relative expression is seen in cercaria. Unlike SmCalp2, the relative expression of SmCalp1 is high in eggs. Figure 5C shows that the expression of SmCalp1 is ~20 fold higher relative to that of SmCalp2 in adult males.

### SmCalp1 and SmCalp2 are present at the tegumental surface

#### Activity based protein profiling

**Figure 6:**
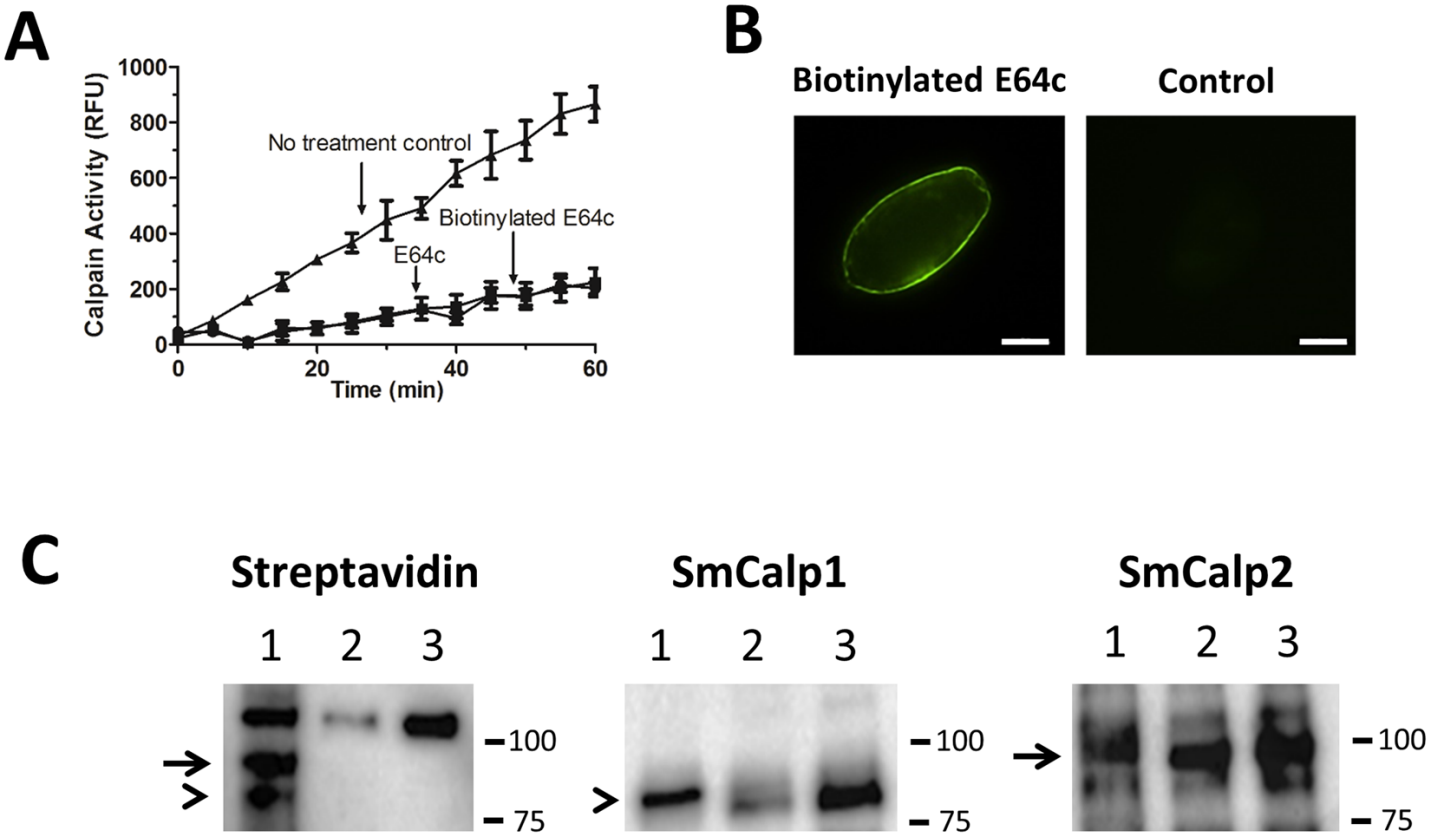
Activity based protein profiling of schistosome tegumental calpains. (**A**) Calpain activity in live adult male worms that were treated with E64c or biotinylated E64c or were untreated. Data are presented as relative fluorescence units (RFU) where fluorescence is derived following substrate cleavage and is measured at excitation/emission 320/480. Two-way ANOVA analysis were used to compare values; values from the no treatment control group differ significantly from either of the E64c treatment groups (p < 0.001, n = 5). (**B**) Localization of biotinylated E64c at the surface of a representative live schistosomulum (left panel); No staining is seen in the absence of biotinylated E64c treatment (Control, right panel). Scale bars represent 50 µm. (**C**) Western blot analysis of extracts of adult worms exposed to biotinylated E64c (lanes 1), E64c (lanes 2) or untreated control (lanes 3) worms. The blot was probed with streptavidin-HRP, (left panel) to detect biotinylated proteins, or with anti-SmCalp1 antibody (center) to detect SmCalp1, or with anti-SmCalp2 antibody (right panel) to detect SmCalp2. SmCalp blots were then probed with HRP-labeled secondary anti-antibody. The arrowhead indicates the position of migration of SmCalp1 and the arrow indicates the position of migration of SmCalp2. A non-specific streptavidin binding protein is detected at the top of all lanes in the “Streptavidin” panel (left). Numbers at right represent the positions of migration of molecular weight markers in kDa.

We have shown that SmCalp1 and SmCalp2 are both expressed in the schistosome tegument and that living worms display clear extracellular calpain activity. In order to determine whether SmCalp1 or SmCalp2 (or both) could be responsible for the activity detected, we employed activity based protein profiling. For this we incubated live parasites with biotin-labeled E64c. This compound (like non-biotinylated E64c) can effectively block the extracellular calpain activity of live worms, as shown in Fig. 6A. Immunostaining using a biotin-binding, streptavidin-Alexa Fluor® 488 conjugate also confirms the presence of biotinylated E64c bound on the parasite surface, as shown in Fig. 6B. A sharp “green ring” on the surface of live schistosomula is evident. In some schistosomula, we additionally see staining in the caecum, probably due to ingestion of the biotinylated reagent.

Extracts of the biotinylated E64c-treated, non-biotinylated E64c-treated and control worms were resolved by SDS-PAGE, blotted to PVDF membrane and subjected to western blot analysis. Probing the blot with a streptavidin-conjugated detection reagent reveals that the extracts of the biotinylated E64c-treated parasites contain proteins of the expected size of both SmCalp1 (Fig. 6C, left panel, lane 1, arrowhead) and SmCalp2 (Fig. 6C, left panel, lane 1, arrow). These two bands are not seen in extracts of control worms treated with non-biotinylated E64c or of untreated controls (Fig. 6C, left panel, lanes 2 and 3). (The >100 kDa molecular weight band seen in all three lanes presumably represents a non-specific streptavidin-binding protein.) Probing equivalent blots with either anti-SmCalp1 antibodies (Fig. 6C, middle panel) or anti-SmCalp2 antibodies (Fig. 6C, right panel) reveals that the position of migration of SmCalp1 (arrowhead) matches that of the lower biotinylated band and the position of migration of SmCalp2 (arrow) matches that of the upper biotinylated band. This result supports the contention that both SmCalp1 and SmCalp2 are accessible for biotinylation from the exterior suggesting that both could be active on the surface of intravascular schistosomes.

### Schistosomes cleave fibronectin

**Figure 7:**
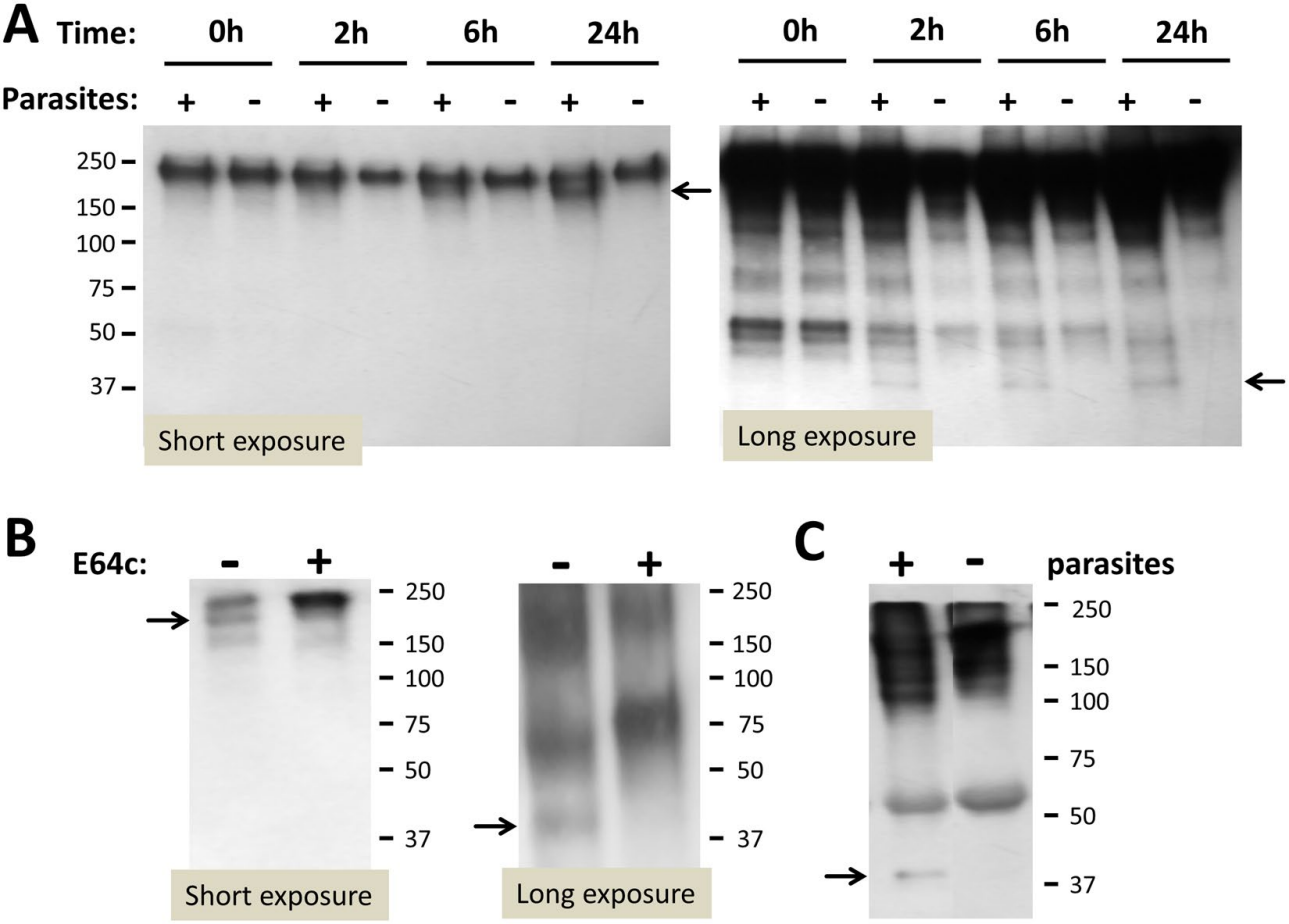
Fibronectin cleavage in the presence of schistosomes. (**A**) Biotinylated fibronectin was incubated in the presence (+) or absence (−) of schistosomula for different time periods (0, 2, 6 or 24 h, as indicated). At each time point aliquots were resolved by SDS-PAGE, blotted to PVDF membrane and probed with streptavidin-HRP. Short exposure of the membrane (left) shows appearance of a fibronectin degradation product (at ~180 kDa, arrow) only in the presence of parasites. Longer exposure (right) shows the concurrent appearance of a ~40 kDa fibronectin degradation product in the same preparations (arrow, right). Numbers at left represent the positions of migration of molecular weight markers, kDa. (**B**) Parasites were incubated with fibronectin in the presence (+) or absence (−) of the non-cell-permeable calpain inhibitor E64c. After 6 h, aliquots were recovered and resolved by SDS-PAGE, blotted to PVDF membrane and probed with streptavidin-HRP. It is clear that the presence of E64c impedes generation of both the~180 kDa high molecular weight (left, arrow) and the ~40 kDa lower molecular weight (right, arrow) fibronectin cleavage products. Numbers at right represent the positions of migration of molecular weight markers, kDa. (**C**) Schistosomula were incubated with mouse plasma for 6 h. An aliquot was recovered and resolved by SDS-PAGE, blotted to PVDF membrane and probed with anti-murine fibronectin antibody. A ~40 kDa band is detected (arrow, left lane) that is not seen in control plasma incubated in the absence of parasites (right lane). Numbers at right represent the positions of migration of molecular weight markers, kDa.

To investigate whether schistosomes could cleave the blood-clotting protein fibronectin, we incubated parasites in the presence of commercially-obtained, pure fibronectin and examined their impact on that protein at 2, 6 and 24 h thereafter. Since the fibronectin used was biotinylated, we detected the full-length protein and any cleavage products at high sensitivity by blotting the samples and probing the blot with a streptavidin conjugate. Figure 7A shows that fibronectin is indeed cleaved in the presence of adult schistosomes. A band running at ~180 kDa (arrow, Fig. 7A left panel) appears beneath the full-length 220 kDa fibronectin monomer and becomes more intense the longer the incubation continues. Longer exposure of this blot reveals an additional cleavage product running at ~40 kDa (Fig. 7A, right panel, arrow) and this product too is generated only in the presence of the parasites. (The longer exposure also reveals that the fibronectin used contains multiple additional biotinylated moieties of diverse molecular weight that can be seen in all lanes, even at 0 h.)

To determine if the cleavage detected could be mediated by surface calpain activity, the experiment was repeated in the presence of the membrane-impermeable calpain inhibitor E64c. As shown in Fig. 7B, it is clear that cleavage is blocked in the presence of the inhibitor (“+” lanes) and that the characteristic cleavage products at ~180 (seen following short exposure, arrow, left panel) and at 40 kDa (seen following long exposure, arrow, right panel) are not detected.

To determine if the parasites can cleave fibronectin in plasma (and not just purified fibronectin), worms were incubated in murine plasma for 6 hours. Aliquots were then resolved by SDS-PAGE, blotted to PVDF membrane and murine fibronectin was detected with a polyclonal anti-fibronectin antibody. In Fig. 7C the pattern obtained is compared with that seen using control plasma that was incubated in the absence of worms. The characteristic ~40 kDa band is only detected in the plasma sample that contained the worms (Fig. 7C, arrow). (Intense staining of high molecular weight moieties prevented the clear detection of the ~180 kDa band in the sample containing worms.)

## Discussion

In this work we focus on the characterization of schistosome tegumental proteases that are expressed at the host-parasite interface. Calpains comprise calcium-dependent cysteine proteases that are widely expressed in nature. Proteomic studies have revealed the presence of two schistosome calpain homologs in the tegument of *Schistosoma mansoni* ^21–23^. We designate these as SmCalp1 and SmCalp2 and we have cloned cDNAs encoding these proteins in this work. The proteins encoded by these cDNAs are predicted to be very similar in size (SmCalp1 is 87 kDa and SmCalp2 is 91 kDa) and the predicted domain structure of both proteins is highly conserved. All domains characteristic of classical calpains are found in SmCalp1 and SmCalp2. These include the protease core domains (PC1 and PC2), the membrane association domain (CBSW) and the C-terminal Ca^2+^-binding penta-EF (PEF) domain. Despite this strong domain conservation, at the amino acid level SmCalp1 and SmCalp2 share just 31% identity. Phylogenetic analysis reveals that each belongs within its own distinct platyhelminth-specific clade. For both SmCalp1 and SmCalp2 we find close homologs in other schistosome species (*S. haematobium* and *S. japonicum*), in the trematode *C. sinensis* and in the cestode *E. granulosus*. It is noteworthy that all members of the calp2 clade have a distinctive catalytic triad, “C, Q, N”, while most all other typical calpains (including the calp1 clade members discussed here) have the traditional catalytic triad “C, H, N”^18^. The presence of glutamine (Q) instead of histidine (H) in the calp2 catalytic triad may be a signature sequence for clade 2 calpains, suggestive of different substrate specificities and inhibition profiles of the different clades. The organization of the SmCalp1 and SmCalp2 genes are also quite distinct. The SmCalp1 gene contains 20 exons and spans over 42 K at the distal end of *S. mansoni* chromosome 1; the SmCalp2 gene contains 12 exons spread over 22 K more centrally on chromosome 1.

Immunofluorescence analysis confirms that SmCalp1 and SmCalp2 are both found in the tegument of the intravascular life stages examined. We see clear peripheral staining in sections of adult male and female worms and in schistosomula.

In order to assess whether the tegumental calpains might be involved in host-parasite interaction (and not simply in internal tegumental metabolism) we incubated live parasites (adult males and females as well as schistosomula) with the non-cell-permeable synthetic peptide “calpain substrate III, Fluorogenic”. All life stages tested were found to be able to cleave this substrate and, given that it is not membrane permeable, we conclude that the activity detected is driven by enzyme that faces the exterior of the parasites. We have no evidence that intravascular schistosomes release or secrete calpains since no activity is detected in either buffer or medium in which worms were previously incubated. Activity is only detected in the presence of living parasites. The activity displayed by a living adult male is ~30% of that detected in an adult male total lysate. This demonstrates that, in addition to being present in the tegument, calpains, not surprisingly, are also well expressed in the internal tissues of the worms. Indeed, the genome of *S. mansoni* contains several calpain homologs beyond SmCalp1 and SmCalp2^15^. Like *S. mansoni*, live adult male worms belonging to the two other schistosome species that are parasitic to humans (*S. japonicum* and *S. haematobium*) also display similar external calpain activity.

The calpain activity displayed by living parasites can be blocked by removing calcium from the reaction buffer or by adding one of several known calpain inhibitors. The inhibitors E64c, calpastatin and PD150606 can each essentially eliminate the surface calpain activity in schistosomula and in adult males. E64c is a non-cell-permeable and irreversible cysteine protease inhibitor^33^ that effectively blocks schistosome surface protease activity but, at least in the short term (1–2 days), has no impact on the morphology of cultured parasites. This suggests that the external calpain does not perform an especially essential function for the parasites in culture. It is only after prolonged exposure to the inhibitor (for 7 days) that schistosomula viability is impacted somewhat, with ~10% greater mortality versus controls. However, we speculate that the schistosome external calpain does play an important role for the parasites within an infected animal, potentially cleaving key host proteins, as described later. Incubating parasites with the related, but membrane permeable, irreversible inhibitor E64d kills all parasites within 24 hours. This shows, not surprisingly, that blocking calpain activity more broadly within schistosomes is catastrophic for the worms.

Developmental expression analysis reveals that both SmCalp1 and SmCalp2 are relatively highly expressed in the parasite’s blood stages, especially in schistosomula and adult males and both are relatively poorly expressed in the cercarial life stage. This suggests that these calpains are of especial importance for the worms in the intravascular environment.

While proteomic analysis of the *S. mansoni* tegument reveals the presence of SmCalp1 and SmCalp2 there, no single proteomic study finds both calpains. SmCalp1 (but not SmCalp2) is reported as being removed from parasites by brief treatment with trypsin (a process called trypsin shaving)^21^ and SmCalp2 (but not SmCalp1) is identified as being available for parasite surface biotinylation^23^. The fact that these proteomic approaches (trypsin shaving and surface biotinylation) detect SmCalp1 and SmCalp2 suggest that these calpains are outward facing and host interactive. To further test this notion here, we utilized activity based protein profiling in which worms were incubated with a biotinylated form of the non-cell-permeable, irreversible inhibitor E64c. This treatment (like treatment of worms with regular, non-biotinylated, E64c) effectively blocks the calpain activity of living parasites. Localization of biotinylated E64c on the treated worms, reveal the reagent bound to their surface. We hypothesize that this surface binding is to external schistosome calpains. Indeed, proteins with the molecular mass of both SmCalp1 and SmCalp2 are biotinylated following this treatment, as revealed by probing western blots of parasite extracts with a (biotin-binding) streptavidin conjugate. This result strongly suggests that both SmCalp1 and SmCalp2 are host exposed in the outer tegument of intravascular schistosomes. This finding is striking since calpains are invariably described as being intracellular^15,16,34^ and our data show that schistosomes display unique, constitutive, functional extracellular calpain activity.

A clue to the normal function of the schistosome extracellular calpain may be the observation that renal ischemia in mice leads to the pathological release of normally intracellular murine calpain into the external milieu where it acts primarily to cleave fibronectin and promote healing^35^. One function of schistosome calpain might be to similarly degrade fibronectin – a 220 kDa protein that plays a central role in generating stable blood clots^36^. Weakening such clots (through calpain-mediated fibronectin cleavage) may be one way that schistosomes prohibit thrombus formation in their vicinity. Certainly, thrombus formation is not detected around the worms *in vivo* ^5,7^ and *ex vivo* the parasites can severely impede the ability of blood to clot^37^. Results reported here show that the parasites in culture can, in fact, cleave added fibronectin to generate ~180 kDa and ~40 kDa fragments. These cleavage fragments are only seen in the presence of parasites but are not generated in the presence of inhibitor E64c. Parasites incubated in murine plasma likewise cleave plasma fibronectin and we conclude that SmCalp1 and/or SmCalp2 is responsible for the fibronectin cleavage observed. In this manner, we hypothesize that schistosomes modulate fibronectin function to limit its ability to contribute to blood clot formation and this permits the worms more unrestricted movement within the vasculature.

Other pathogens are reported to bind fibronectin^37–40^ and culture supernatants of one (the fungus *Cryptococcus neoformans*) is reported capable of cleaving the protein^41^. Our data are the first to show that a metazoan parasite (*S. mansoni*) can likewise target fibronectin for cleavage.

As noted above, our immunolocalization data confirms that both SmCalp1 and SmCalp2 are highly expressed in the intravascular schistosome tegument. These findings corroborate earlier work demonstrating the presence of calpains throughout the tegumental syncytium in *S. mansoni* ^42^ and in the tegument of *S. japonicum* ^43^. Recently, cultured *S. mansoni* adult worms and schistosomula have been shown to release extracellular vesicles and one of the many protein components found therein is SmCalp1^44,45^. SmCalp1 was also shown by proteomic analysis to be among the proteins released during skin invasion by *S. mansoni* cercariae^46^. Likewise the *S. japonicum* homolog, SjCalp1, was demonstrated by immunolocalization to be present in the *S. japonicum* cercarial penetration glands and to be secreted from cercariae^43^. Since fibronectin, in addition to being found in blood, is a key component of the extracellular matrix, it is possible that one function of the secreted SmCalp1 of invading cercariae is to cleave this protein as an aid in the migration of the infecting parasites through the subdermal tissues to the vasculature where the parasites seek to establish a patent infection.

While schistosome tegumental calpains may have more substrates than just fibronectin, one potential explanation for the protective effect of vaccination with SmCalp1 (Sm-p80) is that the immune response generated may block calpain function and prevent the worms from efficiently cleaving fibronectin. Thus migration of cercariae within connective tissue, and migration of blood stage schistosomula and adults within the vasculature, may be severely impeded in vaccinated animals and this may trap and debilitate the worms. Whatever the precise mechanism of action, if an anti-SmCalp1 immune response generates protective immunity, we speculate that targeting the second tegumental calpain, SmCalp2, by vaccination may be additionally beneficial. Since our work confirms that both calpains are accessible to the exterior of the parasites, we suggest that a vaccine targeting SmCalp1 and SmCalp2 together may be an optimal formulation.

## Material and Methods

### Parasites and mice

*Schistosoma mansoni-*infected *Biomphalaria glabrata* snails (strain NMRI) were obtained from the Schistosomiasis Resource Center, at the Biomedical Research Institute (BRI, Cat. No. NR-21962) Rockville MD. Larval schistosomes (cercariae, strain NMRI) were obtained from the infected snails and schistosomula were prepared^47^. Adult male and female parasites were recovered by perfusion from Swiss Webster mice that were infected with 120 cercariae (*S. mansoni*) or 25 cercariae (*S. japonicum*) at BRI, 7 weeks previously. Adult *S. haematobium* were recovered by perfusion of Golden Syrian hamsters that had been infected with 350 cercariae at BRI, 12 weeks previously. All parasites were cultured in complete DMEM/F12 medium supplemented with 10% heat-inactivated fetal bovine serum, 200 U/ml penicillin and 200 µg/ml streptomycin, 0.2 µM Triiodo-L-thyronine, 1 µM serotonin and 8 µg/ml human insulin and were maintained at 37 °C, in an atmosphere of 5%CO_2_^48^. Parasite eggs were isolated from infected mouse liver tissue. All protocols involving animals were approved by the Institutional Animal Care and Use Committees (IACUC) of Tufts University under protocol G2015-113. All experimental procedures were carried out in accordance with approved guidelines of the IACUC.

### Cloning SmCalp1 and SmCalp2

Guided by published sequence^24,25^ (for SmCalp1) and by *S. mansoni* genome sequence online at http://www.genedb.org/Homepage/Smansoni (for SmCalp2), the following primers, that flank the predicted start and stop codons of the calpain cDNAs, were synthesized (IDT Inc, Coralville, IA, USA) and used in a PCR with adult worm cDNA as template: SmCalp1Fw (5′- AAACGCTGTTAAATTGGGTGAACTTTT-3′) and SmCalp1Rv (5′-CTACAGATGCAACCATACTACGACAT-3′), SmCalp2Fw (5′-CTGATCAACCAGGTGAATTTTTATTACGTT-3′) and SmCalp2Rv (5′- AGTTGCAGCTAAACCACTAAGTTCT-3′). PCR conditions were as follows: 50 °C for 2 min, then 95 °C for 10 min; cycling stage (40 cycles), 95 °C for 15 sec, then 60 °C for 2 min. The resulting amplified products were gel purified and sequenced at the Tufts University Core Facility. As described, our SmCalp2 sequence (GenBank accession number: MF590064) differs at the amino terminus compared to that predicted at NCBI (XP_018648578). While a clear SmCalp2 homolog had been annotated in the *S haematobium* genome (accession no: XP_012791984), this was not the case for *S. japonicum*. However, by BLASTing the SmCalp2 coding sequence against the *S japonicum* genome at SchistoDB, one contig (sjc_S000186) was identified. The full SjCalp2 coding DNA was then amplified by PCR using specific primers designed following genome sequence analysis (Accession no: MF590065).

### Anti-SmCalp1 and anti-SmCalp2 antibody production

The peptides NH2-CDGSPQWREISEQEKKN–COOH and NH2-YRLPAGANPPMPRGFFETN-COOH (derived from SmCalp1 and SmCalp2 respectively) were synthesized by Genemed Synthesis, Inc. (San Antonio, TX, USA) and conjugated to bovine serum albumin (BSA). Approximately 500 µg of each peptide-BSA conjugate in Freund’s complete adjuvant was used to immunize two New Zealand white rabbits subcutaneously. The rabbits were boosted with 100 µg of peptide alone in incomplete Freund’s adjuvant 20, 40, and 60 d later. Ten days following this, serum was recovered from the rabbits and anti-SmCalp1 and SmCalp2 antibodies were affinity-purified and dialyzed against phosphate buffered saline (PBS, pH 7.2)^49^.

### Immunolocalization of SmCalp1 and SmCalp2 in *S. mansoni* adult worms and schistosomula

Frozen sections (7 µm thick) of adult parasites embedded in OCT compound were fixed in acetone for 30 min at −20 °C. Cultured schistosomula (7 day) were fixed in 4% paraformaldehyde for 20 min at room temp. Parasites/parasite sections were washed three times in PBS before being blocked with 1% BSA in PBS (blocking buffer) for 1 h. The samples were incubated with either primary anti-SmCalp1 antibody or anti-SmCalp2 antibody at 1:50 dilution for 1 h. After washing with PBST (PBS containing 0.05% Tween-20), parasites were then incubated with Alexfluor-488-anti-rabbit IgG (H + L) (A11034_18913121, Invitrogen, Carlsbad, CA, USA) diluted 1:100 in blocking buffer for 1 h. Samples were washed in PBS and viewed using an inverted fluorescent microscope (TH4–100; Olympus, Tokyo, Japan) equipped with a Retiga 1300 camera (Q Imaging, BC, Canada).

### Calpain activity assay and inhibitor testing

To determine if intact, live parasites expressed calpain activity, living worms (~1000 schistosomula or individual adult male or female worms) were briefly washed and then incubated in calpain assay buffer (20 mM HEPES buffer, pH 7.4, 130 mM NaCl, 1 mM EDTA, 5 mM benzamidine, 10 mM glucose, 0.5 mM phenylmethylsulfonyl fluoride (PMSF), 3 mM CaCl_2_ and 5 mM 2-Mercaptoethanol (2-ME) containing the membrane non-permeable substrate “Calpain substrate III, Fluorogenic” (208771, Calbiochem, Merck KGaA, Darmstadt, Germany, 50 µM)). The peptide sequence of this substrate is: DABCYL-Thr-Pro-Leu-Lys-Ser-Pro-Pro-Pro-Ser-Pro-Arg-EDANS. Calpain activity was monitored by changes in fluorescence arising from substrate cleavage (with excitation at 320 nm and emission at 480 nm, 100 S/TOP) at 25 °C using a Synergy HT spectrophotometer (Bio-Tek Instruments, Winooski, VT, USA).

To investigate the possibility that calpain is released or secreted by cultured parasites, we performed an experiment in which ~1000 schistosomula were first incubated in assay buffer. After 1 hour the buffer was recovered and any calpain activity in the buffer (conditional buffer) was measured. Separately, complete medium in which 1000 schistosomula had been cultured for 3 days (conditional medium) was collected and calpain activity therein was measured. A standard calpain activity assay using 1000 live schistosomula was conducted as a positive control.

Potential inhibitors tested include the non-cell-permeable, cysteine protease inhibitor E64c (Sigma-Aldrich, St. Louis, MO, USA), the cell permeable, uncompetitive calpain inhibitor PD 150606 (Sigma-Aldrich, St. Louis, MO, USA) or the 27-amino acid, cell permeable calpain inhibitor calpastatin (Cat no: 208902, Calbiochem, Merck KGaA, Darmstadt, Germany) which were added to the assay buffer at a final concentration of 100 µM, 100 µM and 2 µm, respectively. The serine protease inhibitor PMSF (0.5 mM), as well as CaCl_2_ (3 mM) and 2-mercaptoethanol (5 mM) were added fresh, at the beginning of each assay. Reactions, in replicate, were started by adding substrate.

To prepare schistosome lysates, adult worms were harvested, washed briefly three times with PBS, and homogenized on ice in 50 μl ice-cold PBS. Calpain activity assays were then conducted in replicate using the amount of homogenate equivalent to one worm.

For the viability assay using inhibitor E64c and its membrane permeable form E64d, 100 µM of each inhibitor was added to 7-day old schistosomula cultured in complete DMEM/F12 medium. Medium plus inhibitor was changed every 2 days. Control schistosomula were cultured minus inhibitor. Parasites were monitored daily and parasite viability was assessed visually. Immobile, granulated parasites were defined as non-viable.

### SmCalp1 and SmCalp2 gene expression analysis

To assess SmCalp1 and SmCalp2 gene expression in different schistosome life stages, RNA was extracted from the parasites using Trizol Reagent (Invitrogen, Carlsbad, USA) following the manufacturer’s instructions. Residual DNA was digested using DNase I (Life Technologies, Carlsbad, USA). cDNA was then synthesized using 1 μg RNA, an oligo-dT primer and Superscript reverse transcriptase III (Invitrogen, Carlsbad, USA). After cDNA synthesis, reverse transcription quantitative PCR (RT-qPCR) was performed using TaqMan Assays, with customized primer sets and reporter probes from Life Technologies (Carlsbad, USA). The following primers and probes were used to detect SmCalp1 and SmCalp2 gene expression. For SmCalp1, primers: forward: 5′- AAACGCTGTTAAATTGGGTGAACTTTT-3′, reverse: 5′-CTACAGATGCAACCATACTACGACAT-3′, probe: 5′-FAM-ACAAGATATCCCTAACTTCC-3′. For SmCalp2, primers: forward: 5′CTGATCAACCAGGTGAATTTTTATTACGTT-3′, reverse: 5′- AGTTGCAGCTAAACCACTAAGTTCT-3′, probe: 5′-FAM-ATGTAGATTCGCAAATTC-3′. As an endogenous control, we used the housekeeping triose phosphate isomerase (TPI) gene, to compare SmCalp1 or SmCalp2 expression across schistosome life cycle stages. Primers used in this analysis were: SmTPI-F, 5′-CATACTTGGACATTCTGAGCGTAGA-3′; SmTPI-R, 5′-ACCTTCAGCAAGTGCATGTTGA-3′; and SmTPI probe, 5′-FAM-CAATAAGTTCATCAGATTCAC-3′. Each RT-qPCR reaction was performed using 1 μl of cDNA, in a final volume of 20 μl. All samples were run in triplicate and underwent 40 amplification cycles on a StepOne Plus system (Life Technologies, Carlsbad, USA). For graphical presentation, values were normalized to males and expressed as percentage difference.

### Activity based protein profiling

Live males were first washed 3 times with Hanks Balanced Salt Solution (HBSS) (Gibco, Waltham, MA, USA), then cultured in serum free DMEM medium. Either E64c or biotinylated E64c (American Custom Chemicals Corporation, San Diego, CA, USA) was added at a final concentration of 100 µM. Control parasites were untreated. After incubation for 30 min at 37 °C, parasites were washed with HBSS 3 times. The surface calpain activity of some parasites was assessed in a standard activity assay, as described above. Some schistosomula were incubated with Alexa Fluor® 488 streptavidin (S11223, Pierce, Waltham, MA, USA at 1:1,00 dilution in PBS, 1% BSA) to localize any biotinylated E64c associated with the parasites. Homogenates of some parasites were processed for SDS-PAGE and western blotting as described next.

### SDS-PAGE and western blot analysis

Protein samples or parasite extracts were resolved by 4–20% SDS-PAGE (BioRad, Hercules, USA), as previously^50^. Proteins were then transferred to PVDF membrane and blocked with TBST (tris-buffered saline pH 7.5, 0.05% Tween 20) containing 5% dry non-fat milk powder for 1 h at room temperature. The membrane was then incubated with primary antibody (Anti-SmCalp1 or anti-SmCalp2 or High Sensitivity Streptavidin- horse radish peroxidase (HRP) conjugate (21130, Pierce, Waltham, MA, USA), as appropriate) for 1 h at room temperature followed by washing with TBST for 30 min and incubation with goat anti-rabbit IgG conjugated to HRP (1:5,000) for 1 h at room temperature. The blots were developed using ECL Detection Reagents (Amersham Bioscience, Piscataway, USA) according to the manufacturer’s instructions and images were recorded using a ChemiDoc™ Imaging System (Bio-Rad).

### Fibronectin cleavage assay

To determine if schistosomes were able to cleave fibronectin, schistosomula (~1,000) or adult parasites (10 males) were first washed with HBSS 3 times, and were then incubated in 300 µl calpain assay buffer containing biotinylated bovine fibronectin (5 ng/µl, FNR03-A, Cytoskeleton, Denver, CO, USA)) at 37 °C. Controls included fibronectin solution without parasites and fibronectin solution containing parasites plus calpain inhibitor E64c (100 µM). Aliquots (30 µl each) were recovered from each group at 0, 2, 6 and 24 h, resolved by SDS-PAGE and fibronectin cleavage products were detected by western blotting as described above, using High Sensitivity Streptavidin HRP conjugate (1:5000).

To assess the ability of parasites to cleave fibronectin in plasma, the experiment just described was repeated using fresh plasma obtained from Swiss Webster mice. Mouse blood was recovered from the tail vein into a collecting tube containing heparin, centrifuged at 13,000 rpm for 15 min at 4 °C, and the supernatant (plasma) was used in this assay. Mouse fibronectin was detected by western blotting using a polyclonal anti-fibronectin antibody (ab23750, Abcam, Cambridge, UK).

### Statistical analysis

For RT-qPCR data, one way analysis of variance (ANOVA) was used and for calpain activity assays, two-way ANOVA was used. P values were considered significant at <0.05. Statistical analyses were performed using GraphPad Prism 5 (La Jolla, CA, USA).

## Electronic supplementary material

Supplementary Information
