# Supplementary material for: The human blood parasite Schistosoma mansoni expresses extracellular tegumental calpains that cleave the blood clotting protein fibronectin
Source: Sci Rep. 2017 Oct 10;7:12912. doi: 10.1038/s41598-017-13141-5 (PMC5635006; doi:10.1038/s41598-017-13141-5)
Supplement: Supplementary file 1 — Supplementary Information [file 41598_2017_13141_MOESM1_ESM.pdf]

Supplementary Information

<u>Manuscript title:</u> The human blood parasite *Schistosoma mansoni* expresses extracellular tegumental calpains that cleave the blood clotting protein fibronectin.

<u>Author list:</u> Qiang Wang, Akram A. Da'dara, Patrick J. Skelly

```
SmCalp1   MG-RIQIVYSPD-----ENVSGRTNRPGKEVV-DPRTGRIIKVKRETPDDYLNVLKPIKG   53
SmCalp2   MQSNPTSNYSMENKLENLNICGNAFGYGHNSSSQYKLRDKTGYPPDTPQKYVNDMRTARH   60

SmCalp1   P-KRMEFNPYL---PKTLTPKGYAKF------------KLMMNVASKQYETLVKRLKTER   97
SmCalp2   NTRAMLFDPERSPLSSKVTENNYCRASPSPVVNRPSTVFSPVGGPSSSFEKIRQDLLNSG   120

SmCalp1   TLWEDPDFPANDKAIGNLPDFRERIEWKRPHEINPNA-----------------KFFAGG   140
SmCalp2   HLFCDSAFPADDSSLYYSQRPPCQIVWMRPSEIVAANSGGGSMGITPRKISTPEFIGEGG   180

SmCalp1   ASRFDIEQGALGDCWLLAVVASISGYPQLFDQVVPKDQELKGPEYVGVVRFRFWRFGHWV   200
SmCalp2   IKLGELRQGELGDCWVVAALAAIASQPSLLSRTIPTGQSFRVEWYAGIFAFRFWRFGRWE   240

SmCalp1   EVLIDDRLPVRQGRNTLVFMHSNDPTEFWSALLEKAYAKLNGCYAHLSGGSQSEAMEDLT   260
SmCalp2   EVYIDDRLPVKSGGQP-LFVHSGRSTEFWPTLLEKAYAKLNGSYEALNVGLVGDAMDDLT   299

SmCalp1   GGICLSLELNQKER-----PSDLIDQLKIYAQRCCLMGCSIDSSVM---EQKMDNGLIGS   312
SmCalp2   GGLTESYTLPGAEDHGLQPPADLDDILIKSFDRRSLITARIKTKGVPGPGFVIPVGFVPG   359

SmCalp1   HAYSLTGVYPVN----YRGRTQWLMRLRNPWGD-SHEWKGAWCDGSPQWREISEQEKKNI   367
SmCalp2   QAFGLTDCRKLRLTDASGSRLLRLVRLRNLWPSVRVDWTGAWSEGSTEWLSLPPQDRLKV   419

SmCalp1   NLSFTADGEFWMSYEDFVTCFSRVEVCHLGLESLEY---NQNFHGKRRLDEAIFSGQWQR   424
SmCalp2   GLV-KAQEEFWMSLEDFIANFDYLDICHLTEPPPVISKSSSAYHSIHNFPSVHLRGRWLR   478

SmCalp1   NVNAGGCINNRTTYWTSPQFRITVEDPDPDDDNKCSVLIGLMQTDIRKK--VGADFQPI   482
SmCalp2   GVSCGGRPYIRASHWANPQFRLVINSPDPNDPDGFATTVIALMQADRRRLRHRAPRLASI   538

SmCalp1   GFMVYNAPDDLNTLLSRAQLLTRSPIAKS-QFINTREVTAQFRVPPGSYVVIPSTFDPNI   541
SmCalp2   GFVLYRLPAGANPPMPRGFFETNHHIASVDFFFDSREVVKRLKLTPGHYLLVPCTYAADQ   598

SmCalp1   EVNFILRVFSQTSI--TEQELDED-----NTNQGLPDDVIEALKLEDTLLDEDQEIEQKF   594
SmCalp2   PGEFLLRLLFDNPDRICESTLERVELSGLAATGPEPDPNYELFQ---------PRLRRLF   649

SmCalp1   LAIRDPKTNAINAVKLGELLNNSTLQ-DIPNFQGFNKELCRSMVASVDNNLTGHVELNEF   653
SmCalp2   Y-EASGESMAVDAFQLEPILNCLLRDDHRAPYSMVSTDACRSLVALLDRDGTGRLCESDF   708
                           1                                  2

SmCalp1   MDLWIQAKGWKHIFIKHDVDQSGYFSAYEFREALNDAGYHVSNRLINAIINRYQDPGTDK   713
SmCalp2   RRVWDILRSWSRLFASFDPQRTAHVTSLDFRIIVEQAGYTLPHTLFSKLVNRFVNVDW-R   767
                                     3                          4
```

```
SmCalp1    ISFEDFMLCMVRLKTAFETIEAHPKNIEGTSLFSAEDYLRFSVYI    758
SmCalp2    LDYPHFISAMSLITKAVAIFKN--FDHGGRAVLPLEQFVEIAMTM    810

                                   5
```

Supplementary figure S1: Alignment of the predicted amino acid sequences of SmCalp1 and SmCalp2 using CLUSTAL. Dashes indicate gaps introduced to maximize matching. Identical amino acids are white on a black background. The catalytic domain (PC1 and PC2), CBSW domain and EF hand domain are indicated with grey (PC1), red (PC2), light blue and green boxes, respectively, corresponding to the diagram in figure 1C. Indicated in red are conserved catalytic residues (C^{154}, H^{313} and N^{337} in SmCalp1 and C^{194}, Q^{330} and N^{296} in SmCalp2). The numbers (1 – 5) below the alignment at the carboxyl end indicate the five EF hand motifs.

```
SmCalp2    -------------------------------------------------MQSNPTSNYSM    11
SjCalp2    -------------------------------------------------MQSNQLLNDSL    11
ShCalp2    -------------------------------------------------MQSNLPLNCSL    11
CsCalp2    MSVSYDPSALSLANLIESPTVPTKSKAMATSRPQVTGLVPGLFESQAALIHSNTKSFY-T    59
EgCalp2    -------------------------------------------------MN--------T    3

SmCalp2    ENKLENLNICGNAFG-------YGHN-SSSQYKLRDKTGYPPDTPQKYVNDMRTARHNTR    63
SjCalp2    ENKLEHLNVCGDDFG-------YSHN-SNSQYRLRDKTGYPPDTPQRYVNDMQTARHNTR    63
ShCalp2    ENKLENLNLCGNSFG-------YGHN-SSSQYKLRDKTGYPPDTPQKYVNDMKTARHNTR    63
CsCalp2    EKEISNILKTVRDMGEVQKLISIAQNSSGADTPLRTVNDFQNRTPTALMNDLEIE-----    114
EgCalp2    NRSL-----------------------KISNSSLNKIGDYPPLRAVSNAN---FGRISET    37

SmCalp2    AMLFDPERSPLSSKVTENNYCRASPSPVV--NRPSTVFSPVGGPSSSFEKIRQDLLNSGH    121
SjCalp2    TMMFDSNIGSLSPKVAENNYCRSSPSPMVA-NRPSTVFSPVGGPSSSFEKIRQELLHSGH    122
ShCalp2    AMLFDSERGPLSPKATENNYCRASPSPMV--NRPSTVFSPVGGPSSSFEKIRQDLLQSGH    121
CsCalp2    ----NRSKSPMHLNLSASNMCRASPSPSVMGSRNNANFIPAGGASSTFEKLRTEALRSGH    170
EgCalp2    DDIFRVSADGQTRFLN---DSGRDSSQMIHDVGPST--KTLPIPSESFEQIRTEHKMLGR    92

SmCalp2    IFCDSAFPADDSSLYYSQRPPCQIVWMRPSEIVAANSGGGSMGITPRKISTPEFIGEGGI    181
SjCalp2    IFCDSAFPADDSSLYYSQRPPCHIVWMRPSEIVAANSGGGSMGITPRRISTPEFIGEGGI    182
ShCalp2    IFCDSAFPADDSSLYYSQRPPCQIVWMRPSEIVAANSGGGSMGITPRKISTPEFIGEGGI    181
CsCalp2    PYSDATFPADDSSLYYSQRPPCHIVWMRPSEIVAAQSGGGLVGIAPRKMSLPEFISEGGA    230
EgCalp2    IFCDSAFPADETSLYYSRRPPCSIVWMRPPEIVSAMCRTDVLGIAPRKIHFPEFIAGGSI    152

SmCalp2    KLGELRQGELGDCWVVAALAAIASQPSLLSRTIPTGQSFRVEWYAGIFAFRFWRFGRWEE    241
SjCalp2    KLSELRQGEL-DCWVVAALAAIASQPSLLSRTIPTGQSFRVEWYAGIFAFRFWRFGRWEE    241
ShCalp2    KLGELRQGELGDCWVVAALAAIAGQPSLLSRTIPTGQSFRVEWYAGIFAFRFWRFGRWEE    241
CsCalp2    KLGELRQGELGDCWVVAALAAISSQPNLLSRIIPVGQSFRPEWYAGIFAFRFWRFGHWEE    290
EgCalp2    RLGDLRQGELGDCWVVAALASMVTQPRLTMRSIPQGQSFRAEWYAGCFCFRFWQFGAWEE    212

SmCalp2    VYIDDRLPVKSGGQPLFVHSGRSTEFWPTLLEKAYAKLNGSYEALNVGLVGDAMDDLTGG    301
SjCalp2    VYIDDRLPVRSGGQPLFVHSGRLNEFWPTLLEKAYAKLNGSYEALNVGLVGDAMDDLTGG    301
ShCalp2    VYIDDRLPVKSGGQPLFVHSGRLTEFWPTLLEKAYAKLNGSYEALNVGLVGDAMDDLTGG    301
CsCalp2    VIIDDRIPVRPGGQPLFVHSGRMTEFWPALLEKAYAKLNGSYEALNVGLVGDAMDDLTGG    350
EgCalp2    VIIDDRLPVRPGGRPLFIHSSRHTEFWPALLEKAYAKLSGSYEALNVGLIGDAMDDIIGG    272

SmCalp2    LTESYTLPGAEDHGLQPPADLDDILIKSFDRRSLITARIKT-------------------    342
SjCalp2    LTESYTLPGAEEQGLQPPSDLDDILIKSFDRRSLITA-----------------------    338
ShCalp2    LTESYTLPGAEDHGLQPPSDLDDILIKSFDRRSLITARIKT-------------------    342
CsCalp2    LTESYTLPAAEEQGILPPPALDDILIKSFDRRSLITARIKV-------------------    391
EgCalp2    LTESYCLAPGEDQGMRPPPDLDDILIKAFDRRSLITARIKASASSSLSGISPSSRLFALT    332

SmCalp2    ------KGVPGPGFVIPVGFVPGQAFGLTDCRKLRLTDASGSRLLRLVRLRNLWPSVRVD    396
SjCalp2    --------PPGPGFVIPAGFVPGQAFGLTDCRKLRLTDASGSRLLRLVRLRNLWPSVRVD    390
ShCalp2    ------KGVPGPGFVIPVGFVPGQAFGLTDCRKLRLTDASGSRLLRLVRLRNLWPSVRVD    396
CsCalp2    ------S-IPGPGFVIPVGFVPGQAFGLTDCRKLRLTDATGSRLVRLVRLRNLWPSVRVG    444
EgCalp2    ALHRRTKGSPCSGFVLPQGFVPGQAFGLTDCRKLRLTDVSGSRLVRLVRLRNLWPSARVG    392
```

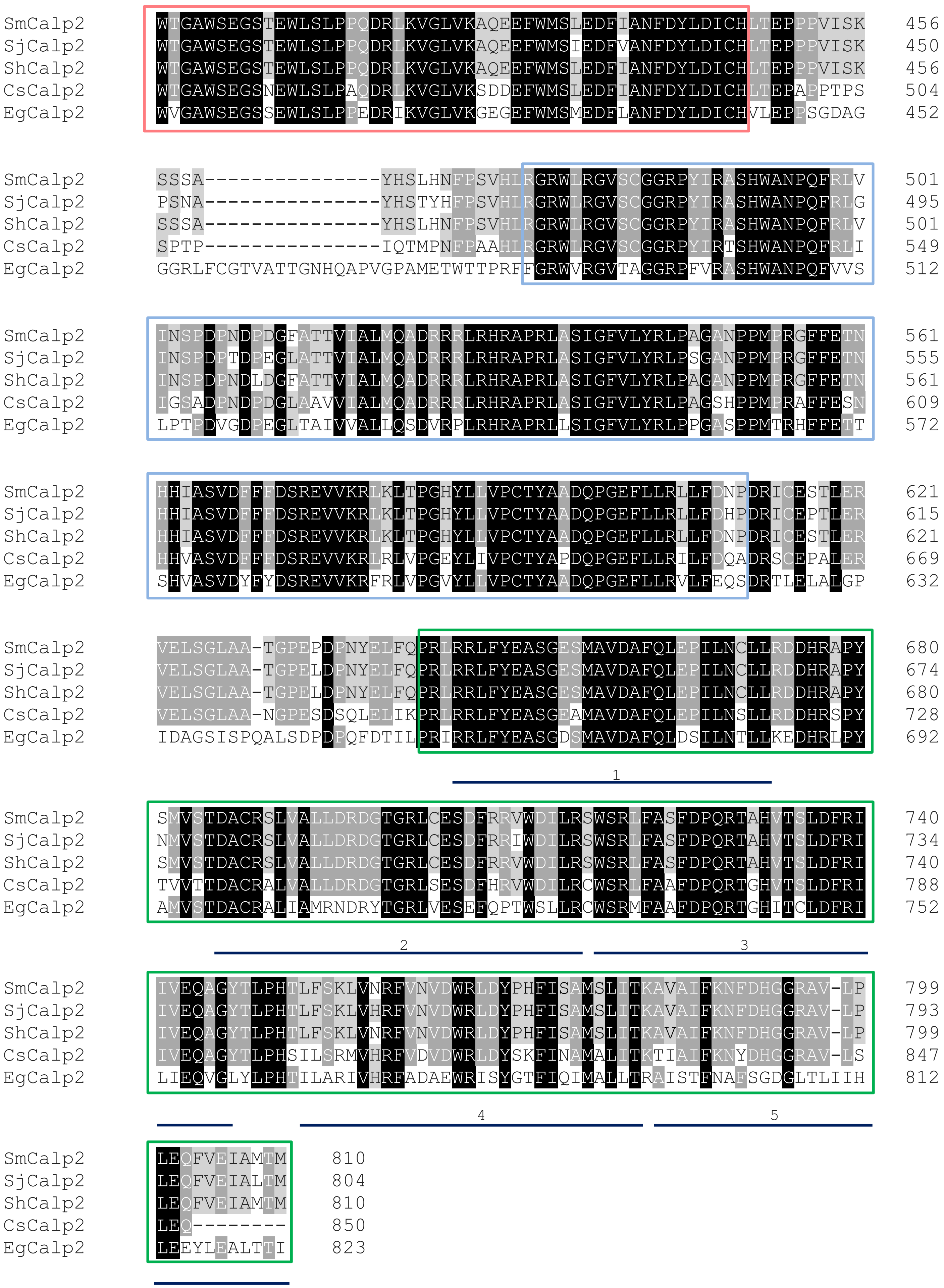
SmCalp2 WTGAWSEGSTEWLSLPPQDRLKVGLVKAQEEFWMSLEDFIANFDYLDICHLTEPPPVISK 456
SjCalp2 WTGAWSEGSTEWLSLPPQDRLKVGLVKAQEEFWMSIEDFVANFDYLDICHLTEPPPVISK 450
ShCalp2 WTGAWSEGSTEWLSLPPQDRLKVGLVKAQEEFWMSLEDFIANFDYLDICHLTEPPPVISK 456
CsCalp2 WTGAWSEGSNEWLSLPAQDRLKVGLVKSDDEFWMSLEDFIANFDYLDICHLTEPAPPTPS 504
EgCalp2 WVGAWSEGSSEWLSLPPEDRIKVGLVKGEGEFWMSMEDFLANFDYLDICHVLEPPSGDAG 452
SmCalp2 SSSA---------------YHSLHNFPSVHLRGRWLRGVSCGGRPYIRASHWANPQFRLV 501
SjCalp2 PSNA---------------YHSTYHFPSVHLRGRWLRGVSCGGRPYIRASHWANPQFRLG 495
ShCalp2 SSSA---------------YHSLHNFPSVHLRGRWLRGVSCGGRPYIRASHWANPQFRLV 501
CsCalp2 SPTP---------------IQTMPNFPAAHLRGRWLRGVSCGGRPYIRTSHWANPQFRLI 549
EgCalp2 GGRLFCGTVATTGNHQAPVGPAMETWTTPRFFGRWVRGVTAGGRPFVRASHWANPQFVVS 512
SmCalp2 INSPDPNDPDGFATTVIALMQADRRRLRHRAPRLASIGFVLYRLPAGANPPMPRGFFETN 561
SjCalp2 INSPDPTDPEGLATTVIALMQADRRRLRHRAPRLASIGFVLYRLPSGANPPMPRGFFETN 555
ShCalp2 INSPDPNDLDGFATTVIALMQADRRRLRHRAPRLASIGFVLYRLPAGANPPMPRGFFETN 561
CsCalp2 IGSADPNDPDGLAAVVIALMQADRRRLRHRAPRLASIGFVLYRLPAGSHPPMPRAFFESN 609
EgCalp2 LPTPDVGDPEGLTAIVVALLQSDVRPLRHRAPRLLSIGFVLYRLPPGASPPMTRHFFETT 572
SmCalp2 HHIASVDFFFDSREVVKRLKLTPGHYLLVPCTYAADQPGEFLLRLLFDNPDRICESTLER 621
SjCalp2 HHIASVDFFFDSREVVKRLKLTPGHYLLVPCTYAADQPGEFLLRLLFDHPDRICEPTLER 615
ShCalp2 HHIASVDFFFDSREVVKRLKLTPGHYLLVPCTYAADQPGEFLLRLLFDNPDRICESTLER 621
CsCalp2 HHVASVDFFFDSREVVKRLRLVPGEYLIVPCTYAPDQPGEFLLRILFDQADRSCEPALER 669
EgCalp2 SHVASVDYFYDSREVVKRFRLVPGVYLIVPCTYAADQPGEFLLRVLFEQSDRTLELALGP 632
SmCalp2 VELSGLAA-TGPEPDPNYELFQPRLRRLFYEASGESMAVDAFQLEPILNCLLRDDHRAPY 680
SjCalp2 VELSGLAA-TGPELDPNYELFQPRLRRLFYEASGESMAVDAFQLEPILNCLLRDDHRAPY 674
ShCalp2 VELSGLAA-TGPELDPNYELFQPRLRRLFYEASGESMAVDAFQLEPILNCLLRDDHRAPY 680
CsCalp2 VELSGLAA-NGPESDSQLELIKPRLRRLFYEASGEAMAVDAFQLEPILNSLLRDDHRSPY 728
EgCalp2 IDAGSISPQALSDPDPQFDTILPRIRRLFYEASGDSMAVDAFQLDSILNTLLKEDHRLPY 692
1
SmCalp2 SMVSTDACRSLVALLDRDGTGRLCESDFRRVWDILRSWSRLFASFDPQRTAHVTSLDFRI 740
SjCalp2 NMVSTDACRSLVALLDRDGTGRLCESDFRRIWDILRSWSRLFASFDPQRTAHVTSLDFRI 734
ShCalp2 SMVSTDACRSLVALLDRDGTGRLCESDFRRVWDILRSWSRLFASFDPQRTAHVTSLDFRI 740
CsCalp2 TVVTTDACRALVALLDRDGTGRLSESDFHRVWDILRCWSRLFAAFDPQRTGHVTSLDFRI 788
EgCalp2 AMVSTDACRALIAMRNDRYTGRLVESEFQPTWSLLRCWSRMFAAFDPQRTGHITCLDFRI 752
2
3
SmCalp2 IVEQAGYTLPHTLFSKLVNRFVNVDWRLDYPHFISAMSLITKAVAIFKNFDHGGRAV-LP 799
SjCalp2 IVEQAGYTLPHTLFSKLVHRFVNVDWRLDYPHFISAMSLITKAVAIFKNFDHGGRAV-LP 793
ShCalp2 IVEQAGYTLPHTLFSKLVNRFVNVDWRLDYPHFISAMSLITKAVAIFKNFDHGGRAV-LP 799
CsCalp2 IVEQAGYTLPHSILSRMVHRFVDVDWRLDYSKFINAMALITKTIAIFKNYDHGGRAV-LS 847
EgCalp2 LIEQVGLYLPHTILARIVHRFADAEWRISYGTFIQIMALLTRAISTFNAFSGDGLTLIIH 812
4
5
SmCalp2 LEQFVEIAMTM 810
SjCalp2 LEQFVEIALTM 804
ShCalp2 LEQFVEIAMTM 810
CsCalp2 LEQ-------- 850
EgCalp2 LEEYLEALTTI 823

Supplementary figure S2: Alignment of the predicted amino acid sequences of the platyhelminth Calp2 clade 2 proteins using CLUSTAL. Dashes indicate gaps introduced to maximize alignment. Amino acids identical in all five sequences are depicted in white with a black background, amino acids found in 4/5 have a grey background and in 3/5 have a light grey background. The catalytic domain (PC1 and PC2), CBSW domain and EF hand domain are indicated with grey (PC1), red (PC2), blue and green boxes, respectively. Red amino acids indicate conserved catalytic residues. The numbers (1 – 5) below the alignment at the carboxyl end indicate the five EF hand motifs. SmCalp2, *S. mansoni* Calp2 (SmCalp2; MF590064); SjCalp2, *S. japonicum* Calp2 (SjCalp2, MF590065)**;** ShCalp2, *S. haematobium* Calp2 (ShCalp2, XP_012791984) CsCalp2, *Clonorchis sinensis* Calp2 (Cs2, GAA29557.2); EgCalp2, *Echinococcus granulosus* Calp2 (Eg2, CDS20315.1).

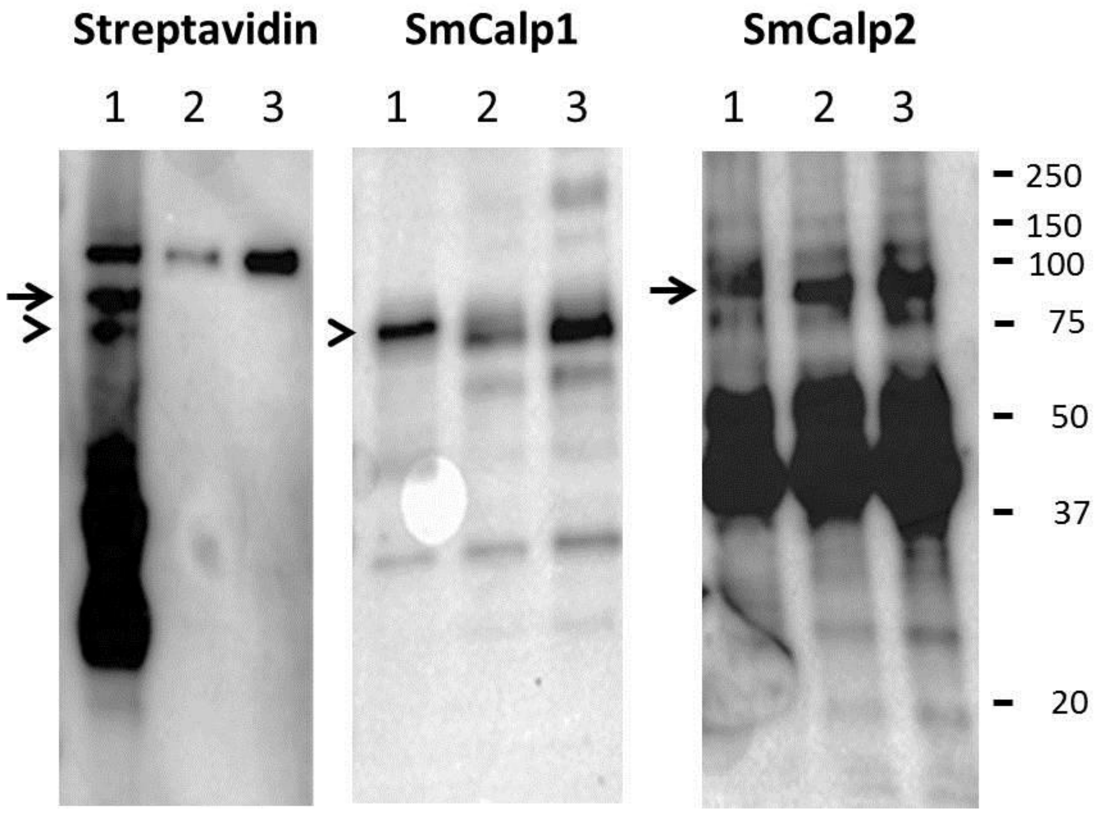

Supplementary figure S3: The entire blot from figure 6C - western blot analysis of extracts of adult worms exposed to biotinylated E64c (lanes 1), E64c (lanes 2) or untreated control (lanes 3) worms. The blot was probed with streptavidin-HRP, (left panel) to detect biotinylated proteins or with anti-SmCalp1 antibody (center) to detect SmCalp1 or with anti-SmCalp2 antibody (right panel) to detect SmCalp2. Blots were then probed with HRP-labeled secondary anti-antibody. Lanes 1 contain extracts of biotinylated E64c-treated worms, lanes 2 contain extracts of E64c-treated worms, lanes 3 contain extracts of untreated, control worms, The arrowhead indicates the position of migration of SmCalp1 and the arrow indicates the position of migration of SmCalp2. A non-specific streptavidin binding protein is detected at the top of all lanes in the "Streptavidin" panel (left). Some of the additional lower molecular weight, biotinylated moieties seen in "Streptavidin" lane 1 (left) may represent biotinylated SmCalp2 fragments, also detected by the anti-SmCalp2 antibody in "SmCalp2" lanes 1, 2 and 3 (right panel).
